# Utility of Zinc Oxide Nanoparticles Catalytic Activity in the Electrochemical Determination of Minocycline Hydrochloride

**DOI:** 10.3390/polym12112505

**Published:** 2020-10-28

**Authors:** Amal M. Al-Mohaimeed, Wedad A. Al-Onazi, Maha F. El-Tohamy

**Affiliations:** Department of Chemistry, College of Science, King Saud University, P.O. Box 22452, Riyadh 11495, Saudi Arabia; walonazi@ksu.edu.sa (W.A.A.-O.); moraby@ksu.edu.sa (M.F.E.-T.)

**Keywords:** minocycline hydrochloride, potentiometric sensors, zinc oxide nanoparticles

## Abstract

The current work described the synthesis and characterization of zinc oxide nanoparticles (ZnONPs) and their electrocatalytic activity in the determination of minocycline hydrochloride (MCL). The unique features of metal oxide nanoparticles such as zinc oxide encourage the researchers to investigate the activity of metal oxide nanoparticles as remarkable semiconductor materials active in the electrochemical sensing determination. Herein, the suggested study displayed a comparative determination of minocycline hydrochloride using two conventional and modified ZnONPs-coated wire sensors. The recorded results showed the linear behavior of the enriched ZnONPs sensor over the 1.0 × 10^−10^–1.0 × 10^−2^ mol L^−1^ with respect to 1.0 × 10^−6^–1.0 × 10^−2^ mol L^−1^ for the conventional sensor. The two sensors are working in the pH range of 3–5 with regression equations E_mV_ = (53.2 ± 0.5) log [MCL] + 448.8 and E_mV_ = (58.7 ± 0.2) log [MCL] + 617.76 for conventional and enriched ZnONPs, respectively. The correlation coefficients were 0.9995 and 0.9998 for the previously mentioned sensors, respectively. The validity of the suggested analytical method was evaluated according to the recommended guidelines for methodology and drug analysis. The developed sensors were also used in the quantification of MCL in commercial formulations.

## 1. Introduction

Nanoengineered metal oxide semiconductors are considered as excellent materials for the construction of highly selective and sensitive electrochemical sensors and biosensors [1,2]. Zinc oxide (ZnO) with a wurtzite crystal structure is an n-type semiconductor with a high isoelectric point (IEP, 9.5), wide band gap (3.2 eV), and high electron communication features [3]. The unique physical and chemical properties of zinc oxide nanoparticles (ZnONPs), including a large specific surface area, ability of strong adsorption, high catalytic efficiency, and biocompatibility, has introduced a new offshoot to electrochemical science [4]. Furthermore, due to their specific optical, electrical, and catalytic potentials ZnONPs are very important materials for tremendous applications such as solar cells [5], sensors [6], water splitting [7], and biomedical applications [8]. Different production techniques have been utilized for diverse ZnONPs structures, e.g., highly controlled structures through electron beam lithography, rough nanomorphology using radiofrequency sputtering, three-dimensional structures using printing technologies, nanofibers through electrospinning, and different wet chemistry methods [9,10,11,12,13].

Potentiometric sensors typically are composed of four main parts: a power source, a transducer, a selective membrane layer for analyte recognition and an electronic signaling system. Potentiometric chemical sensors for the detection of pharmaceutical compounds, metals, or organic species are the classic example of self-powered sensors that do not require any external energy source for their operation, because their measuring systems are based on two electrodes: a working and standard reference electrode, and they can use the chemical energy available in the solution [14]. The potentiometric measurements in self-powered sensors are conducted by the accumulation of analytes under the electrostatic mechanism, leading to the formation of a potential difference between the surface of the working electrode and the reference one [14]. The most two important points in the development of a high efficient sensor are the electroactive materials used and the fabrication design. The diverse structure and size of synthesized ZnONPs provides an exceptional capability for the fabrication of catalytic potentially sensing systems with fast and ultrasenstive and selective features such as wide concentration ranges, low detection limits, strong reproducibility, higher percentage recoveries, and the ability to work under room-temperature conditions [15]. The literature review showed many addressed articles concerned with the important role of ZnONPs in electrochemical sensors [16,17,18].

Potentiometric sensors with ionophores (ion pairs) bases on polymer membranes can determine various dozens of analytes. Polyvinyl chloride (PVC) is the most commonly applied polymer. Esters of organic acids are usually used as plasticizers, such as dibutyl sebacate (DBS), di octyl sebacate (DOS), di butylphthalate (DBP), etc. In addition, some ethers e.g., *o*-nitrophenyl octyl ether (*o*-NPOE) act as solvent mediators for ion pairs. Ionophores (ion pairs) are lipophilic molecules or ions capable of forming a specific interaction with other ions in the membrane, which predetermines the selectivity of the corresponding sensor [19]. Phosphomolybdic acid (PMA) or (dodeca molybdophosphoric acid) is a yellow to greenish chemical compound that is freely soluble in water and polar solvents. It is commonly used in potentiometric studies due to its capability to interact with many different analytes [20,21].

In the coated wire sensor type, a metal wire (Al, Cu, Pt, Au, or Ag) is used as a sensor substrate and the polymer membrane cocktail is deposited on the surface of the selected wire. The sensors of coated wire nature are simple in design, fast in potential response, mechanically stable, reproducible, and usually exhibit excellent selectivity rather than the conventional liquid membrane type [22].

Minocycline hydrochloride (MCL) is recommended to treat various bacterial infections, such as urinary, respiratory, and skin infections. It may also be used with other medications to treat severe acne. This medication belongs to a class of drugs known as tetracycline antibiotics. It works by stopping the growth of bacteria [23].

Several analytical techniques were previously reported for the determination of MCL including spectroscopic methods and [24,25] chromatographic separation [26,27,28,29]. Additionally, a few electrochemical methods were addressed for the determination of MCL [30,31].

This study aims to synthesize ZnONPs using a simple sol–gel preparation method. Different spectroscopic and microscopic techniques were used to confirm the formation of ZnONPs. The synthesized nanoparticles were used as a catalytic metal oxide nanofilm in the fabrication of a modified coated wire potentiometric minocycline–phosphomolybdate (MCL-PM) sensor for the detection of minocycline hydrochloride in its commercial products. A comparative study was performed between the modified sensor and the conventional type toward the slope, sensitivity, selectivity, detection limits, and the range of quantification of the investigated drug.

## 2. Experimental

### 2.1. Chemicals and Instruments

Pure minocycline hydrochloride and zinc acetate were provided by Saudi Pharmaceutical Industries & Medical Application Corporation (SPIMACO), Al-Qassim pharmaceutical Plant, Al-Qassim, Saudi Arabia. Commercial tablets of (Minocin 50 mg/tablet) were purchased from local drug stores (Riyadh, Saudi Arabia). Other chemicals and solvents such as ortho-nitrophenyloctyl ether (*o*-NPOE), high molecular weight polyvinyl chloride (PVC), zinc acetate dihydrate (Zn(CH_3_CO_2_)_2_·2 H_2_O, 99.0%), phosphomolybdic acid (PMA), acetone 99.9%, ethanol 99.9% tetrahydrofuran (THF) 97.0%, and methanol 99.9% was supplied from Sigma Aldrich, Hamburg, Germany.

Potentiometric measurements were performed using a digital pH/mV (HANNA, model 211, HANNA Instrumennts, Winsocket, RI, USA) with a modified indicator electrode minocycline–phosphomolybdate–zinc oxide nanoparticles (MCL/PM/ZnONPs) in conjunction with double junction Ag/AgCl. A pH meter Metrohm model 744 (Metrohm Co., Herisau, Switzerland) was used to adjust the pH throughout all measurements.

### 2.2. Synthesis of Zinc Oxide Nanoparticles

The synthesis of ZnONPs was performed using the sol–gel method [32] with slight modification. Briefly, 6 g of zinc acetate dihydrate (Zn(CH_3_CO_2_)_2_·2H_2_O) was dissolved under continuous magnetic stirring in 200 mL of distilled water at room temperature for 2 h. The obtained solution was heated to 50 °C, and 300 mL of absolute ethanol was added under continuous magnetic stirring. Consequently, 3.0 mL of mol L^−1^ of sodium hydroxide (0.2 mol L^−1^) was added dropwise under the same magnetic stirring to form a clear solution. The obtained solution was left aside for one day. A white precipitate was formed; then, it was filtered and washed three times with ethanol and dried at 80 °C for 60 min. The overall chemical reaction to prepare ZnONPs when sodium hydroxide was used as solvent is stated as follows:

$$\mathrm{Zn}({\mathrm{CH}}_{3}\mathrm{COO}{)}_{2}.2H_{2}O\;+\;2\mathrm{NaOH}\;\to \;\mathrm{ZnO}\;+\;2{\mathrm{NaCH}}_{3}\mathrm{COO}\;+\;H_{2}O.$$

**Figure 1:**
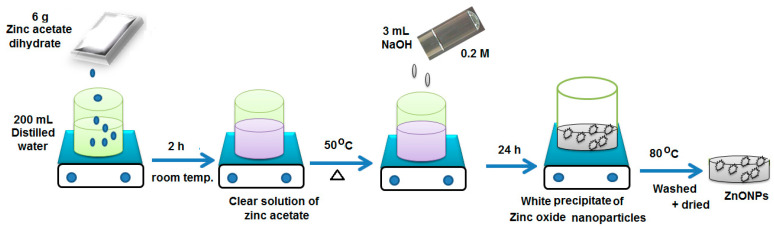
Sol–gel synthesis of zinc oxide nanoparticles.

Figure 1 shows the sol–gel synthesis of zinc oxide nanoparticles.

### 2.3. Characterization of Nanoparticles

Spectral analysis of the prepared ZnONPs was recorded using a UV 2450 Spectrophotometer (Shimadzu Corporation, Kyoto, Japan) in the range of 200–600 nm. Fourier-Transform Infrared spectroscopy (FT-IR) analysis was also carried out using the Spectrum BX spectrometer, (PerkinElmer, Waltham, MA, USA) to confirm the possible functional groups present in the synthesized ZnONPs. X-ray diffraction (XRD) (Shimadzu XRD-6000 diffractometer, Kyoto, Japan) was used to determine the crystallographic structure of the formed nanoparticles. A microscopic study was carried out using a transmission electron microscope (TEM) (JEM-2100F, JEOL Ltd., Akishima, Tokyo, Japan) to characterize the particle size of ZnONPs under an applied accelerating voltage of 100 kV and ×25000 magnification. In addition, scanning electron microscope (SEM), JEOL JSM-6060 LV model (Akishima, Tokyo, Japan) was used to characterize the surface morphology of the prepared ZnONPs. Energy-Dispersive X-ray Spectroscopy (EDX) analysis was conducted using an SEM microscope (JSM-7610F; JEOL Akishima, Tokyo, Japan) equipped with EDX to confirm the existence of zinc and oxygen elements in the prepared samples.

### 2.4. Preparation of Stock Minocycline Hydrochloride Solution

An accurate amount of MCL drug equivalent to prepare 1.0 × 10^−2^ mol L^−1^ was prepared by dissolving 0.53 g of MCL in 100 mL of distilled water. Different analytical solutions were prepared using the same solvent.

### 2.5. Preparation of Ion Pair

The ion pair minocycline–phosphomolybate (MCL-PM) was prepared by mixing 20 mL of each 1.0 × 10^−2^ mol L^−1^ of MCL and PMA aqueous solutions. The mixture was stirred under magnetic stirring for 10 min. A pale yellowish precipitate of MCL-PM was formed. The produced precipitate was filtrated off using a Whatman filter paper No. 40, washed with deionized water, and dried overnight at ambient room temperature. Then, the obtained ion pair was finally ground and used for further preparation of a coated membrane cocktail [33].

### 2.6. Sensor Construction

**Figure 2:**
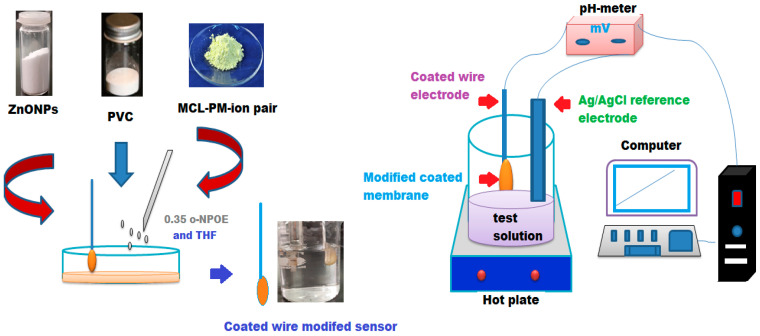
Illustrated the construction of the modified sensor and its potentiometric system.

The coated plastic membrane cocktail is usually formed from high molecular weight polyvinyl chloride (PVC, 33%), ion pair 1.5%, and 65% plasticizer in the presence of organic solvent THF. Two conventional and modified coated wire sensors were constructed using a coated membrane containing a coated plastic membrane cocktail prepared by mixing PVC (190 mg), an MCL-PM ion pair (10 mg), and 0.35 mL of plasticizer *o*-NPOE in 5 mL of THF. The mixture was left for 1 h at room temperature for slow evaporation to obtain an oily viscous cocktail. Before constructing the sensor, a pure Al wire was cleaned with acetone and dried with tissue paper; then, it was immersed in the coating mixture several times to obtain a coating membrane layer. Then, it was left to dry slowly at room temperature for one day. Finally, the constructed sensors were preconditioned in drug solution (1.0 × 10^−3^ mol L^−1^). The formed sensor was assembled as follows: Al wire/coated membrane/test solution/Ag/AgCl reference electrode. To construct the modified sensor, a plastic membrane cocktail containing ZnONPs (5 mg), PVC (190 mg), an MCL-PM ion pair (10 mg), and 0.35 mL of plasticizer *o*-NPOE in 7 mL of THF was prepared. The mixture was continuously stirred for 15 min at room temperature until the formation of a homogenoeus well-dispersed membrane mixture. A polymeric MCL-PM-ZnONPs-coated membrane mixture was used to form a thin layer on the surface of the sensor; then, after drying, the sensor was immersed several times in the coated membrane mixture to form a thick-coated wire membrane. The formed sensor was assembled as follows: Al wire/modified-coated membrane/test solution/Ag/AgCl reference electrode. Figure 2 illustrates the construction of the modified sensor and its potentiometric system.

### 2.7. Calibration Graphs

The calibration graphs of conventional MCL-PM and modified MCL-PM-ZnONPs-coated wire sensors were constructed by plotting the sensor potentials/mV against a logarithm of MCL concentrations (1.0 × 10^−10^–1.0 × 10^−2^ mol L^−1^). The electrochemical measurements were carried out by measuring 50 mL of the tested drug solution by the as-prepared working sensors in conjunction with the Ag/AgCl reference electrode.

### 2.8. Factors Affecting the Potential Readings

Three main factors affecting the potential reading were studied. The effect of pH was tested using 1.0 × 10^−4^ mol L^−1^ of MCL test solution, and the potential readings of the constructed sensors were recorded. The constructed sensors in conjunction with combined glass and Ag/AgCl reference electrodes were immersed separately in 50 mL of MCL 1.0 × 10^−4^ mol L^−1^. To determine the safe pH range of the constructed sensor, hydrochloric acid and NaOH (1.0 × 10^−2^ mol L^−1^) was added gradually to acidify and then elevate the pH, respectively. The pH graph was plotted using the pH values vs. the potential readings [34].

The selectivity of the constructed sensors was tested using a separate solution method [35]. The potentiometric selectivity coefficient of the constructed sensors was evaluated using separate solutions of each 1.0 × 10^−3^ mol L^−1^ MCL and interfering species. The selectivity of the conventional and modified sensor was measured in the presence of various possible interfering species such as cations (Na^+^, K^+^, Ag^+^, Ni^2+^, Cu^2+^, Zn^2+^, Mg^2+^, and Fe^3+^), sugars (lactose, glucose), and amino acids (L-histidine, ornithine, and glycine). In addition, the selectivity of the sensors was investigated in the presence of additive compounds, which are used in the formation of MCL commercial tablets including talc, silicon dioxide (SiO_2_), titanium dioxide (TiO_2_), magnesium stearate, and microcrystalline cellulose. The selectivity coefficient was calculated using the following equation:

Log K^pot^ = (E_2_ − E_1_)/S + Log [Drug] − Log [B^z+^]^1/z^

where K^pot^, E_1_, E_2_, B^z+^, and S are the selectivity coefficient, the electropotential of 1.0 × 10^−3^ mol L^−1^ MCL solution, the electrode potential of 1.0 × 10^−3^ mol L^−1^ of interfering species, interfering ions, and the slope of the calibration graph, respectively.

### 2.9. Analytical Applications

Ten individual tablets of minocycline hydrochloride^®^ tablets (50 mg/tablet) were finely powdered, and an accurate amount suitable to obtain 1.0 × 10^−2^ mol L^−1^ standard solution was dissolved in 50 mL of distilled water. Then, the solution was filtered, and the filtrate was completed to 100 mL by the same solvent. Analytical solutions (1.0 × 10^−2^–1.0 × 10^−10^ mol L^−1^) were prepared and the developed modified MCL-PM-ZnONPs sensor was applied to determine each concentration of the tested MCL solution.

## 3. Results and Discussion

### 3.1. Characterization of ZnONPs

**Figure 3:**
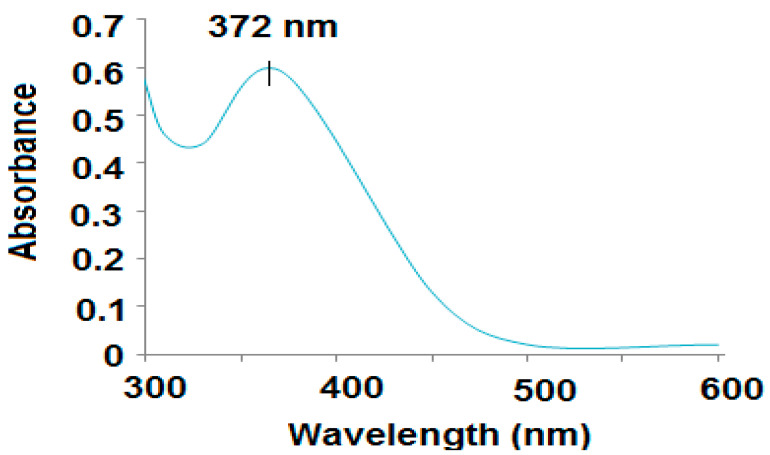
UV spectrum of zinc oxide nanoparticles, the absorption was measured at a wavelength of 200–600 nm.

Different spectroscopic techniques, including UV-Vs, FT-IR, XRD, and EDX analysis were applied to characterize the sol–gel synthesized ZnO nanoparticles. UV-Vis spectroscopy is one of the most useful and reliable techniques suitable for characterizing and revealing the size, shape, and stability of the prepared zinc oxide nanostructures in its aqueous suspension [36]. The UV-Vis spectrum of ZnONPs exhibited a broad absorption peak at 372 nm (Figure 3). This broad peak can be due to the photoexcitation of electrons from the valence band to the conduction band. The optical band gap energy (Eg) can be determined from the absorption coefficient (A) using the Tauc relation: Eg = hυ = hc/λ, where h is Planck’s constant, c is the velocity of light, and λ is the wavelength. The band gap of ZnONPs was found to be 3.16 eV. The band gap values confirmed that these nanoparticles are semiconductors, and the results are in agreement with those previously reported [37].

**Figure 4:**
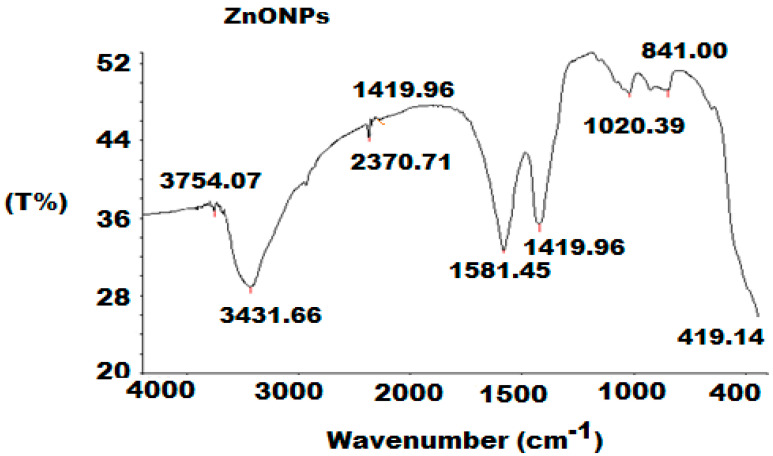
Fourier-Transform Infrared spectroscopy (FT-IR) spectrum of zinc oxide nanoparticles (ZnONPs) at a wavenumber from 4000 to 400 cm^−1^.

The FT-IR spectrum of the prepared ZnONPs exhibited distinct bands at 3754.07 cm^−1^ and 3431.66 cm^−1^, which were related to the OH-stretching groups. Whereas, two absorption bands at 2340.65 cm^−1^ and 2370.71 cm^−1^ were observed, confirming the presence of CH-stretching vibrations. The bands at 1581.45 cm^−1^ and 1419.96 cm^−1^ were assigned to be corresponding to a C=O group of carboxylic acid. The peak of 1020.39 cm^−1^ is related to the CO group of acetate, and the peak assigned at 419.14 cm^−1^ confirmed the formation of Zn metal stretching (Figure 4).

**Figure 5:**
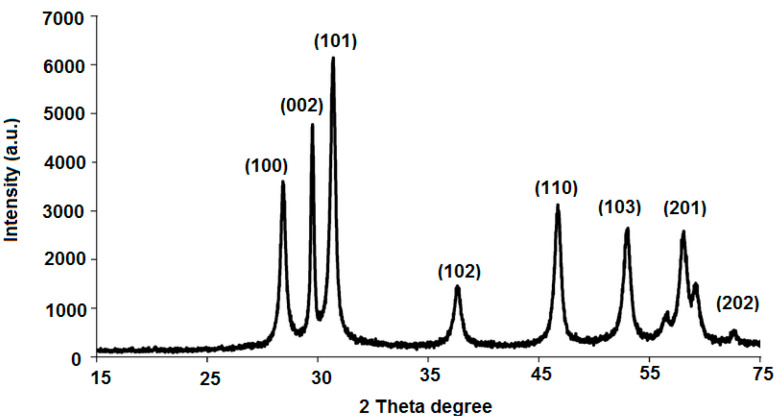
XRD pattern of sol–gel synthesized zinc oxide nanoparticles.

The crystalline phase and size of the as-prepared ZnONPs was investigated by carrying out XRD analysis using a Shimadzu 6000 diffractometer equipped with a Cu kα (K = 1.54 Å) source, an applied voltage of 40 kV, and a current at 30 mA. The XRD morphology of the as-prepared sol–gel ZnONPs displayed X-ray diffraction peaks at angles (2θ) of 32.41°, 34.63°, 36.82°, 47.56°, 56.32°, 64.12°, 68.24°, and 72.82° correspond to the reflection from (100), (002), (101), (102), (110), (103), (201), and (202) crystal planes (Figure 5). All XRD peaks of the prepared nanoparticles related to the hexagonal wurtzite structure of the ZnONPs property (*a* = 0.315 nm and *c* = 0.529 nm) [38].

**Figure 6:**
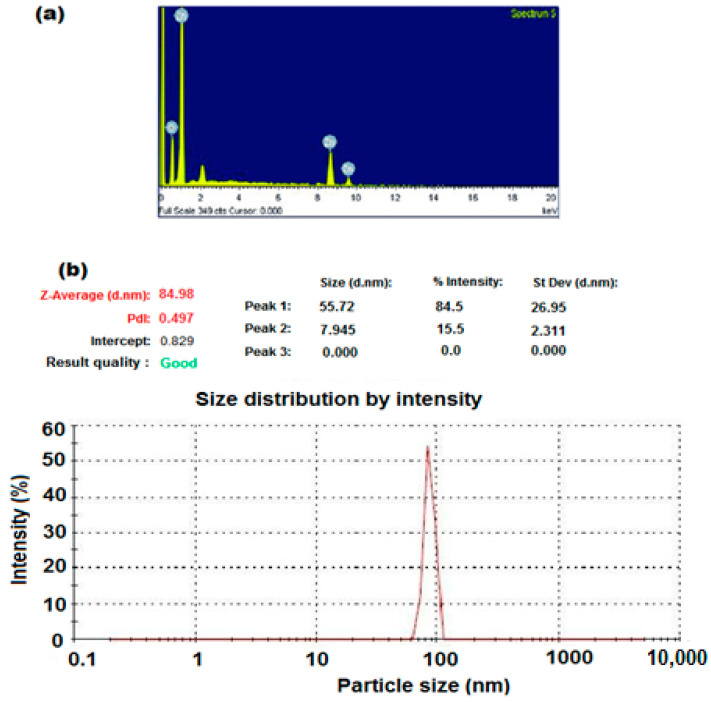
(**a**) Energy-Dispersive X-Ray Spectroscopy (EDX) of ZnONPs and (**b**) Particle size analyzer for ZnONPs using sol–gel method and zinc acetate as precursor.

The EDX characterization spectrum of ZnONPs (Figure 6a) suggested that the content of zinc is 84.3% and the oxygen content is 15.7%. The results showed that the main constituents are Zn and O, revealing good purity, and no trace impurities were observed within the limit of detection of EDX [39]. The particle size of the formed ZnONPs was estimated using a particle size analyzer (Figure 6b). The obtained results confirmed that the formed ZnONPs are in nanoscale form and were found to be 85.92 nm. The obtained results are attributed to the length of the particle structure. The recorded results are in agreement with the XRD results that revealed that the synthesized ZnONPs exhibit an excellent crystalline form. Furthermore, the obtained results were in agreement with previously prepared ZnONPs by Ossai et al. [40].

**Figure 7:**
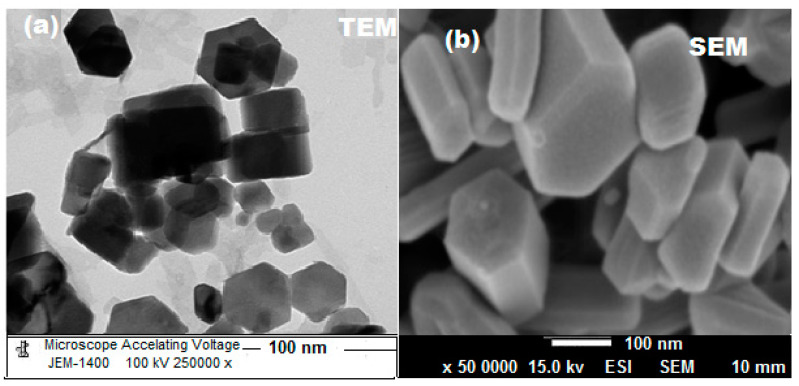
(**a**) Transmission electron microscope (TEM) and (**b**) Scanning electron microscope (SEM) images of ZnONPs.

Microscopic techniques were carried out to characterize the formed ZnONPs. The picked images of TEM showed that the prepared ZnONPs are uniformly distributed and hexagonal in shape with a particle size of 80–100 nm (Figure 7a). In addition, the surface morphology of the synthesized ZnONPs was confirmed by SEM analysis (Figure 7b).

### 3.2. The Nature of the Suggested Sensors

The potentiometric performance characteristics of MCL-PM sensors are based on the formation of a stable complex due to the incorporation of MCL with PMA to form an MCL-PM electroactive species that selectively binds with the MCL ions of interest. The formed electroactive ion pair is insoluble in water but readily soluble in organic solvents such as tetrahydrofuran (THF). As it is well known, the PVC electroactive selective membrane required the use of a plasticizer as a solvent mediator [41]. In the current study, ortho-nitrophenyloctyl ether (*o*-NPOE) was mixed with the active material MCL-PM in the presence of PVC to produce the coated wire material. The *o*-NPOE acts as a fluidizer, helping the homogenous dissolution of the ion pair and allowing its diffusion mobility inside the membrane. The high dielectric constant of *o*-NPOE (ε = 24) improves the membrane selectivity toward the tested analyte by affecting the dissolution of the ion pair within the active membrane and consequently increasing its partition coefficient in the prepared membrane, giving the suitable mechanical feature of the membrane [42].

**Figure 8:**
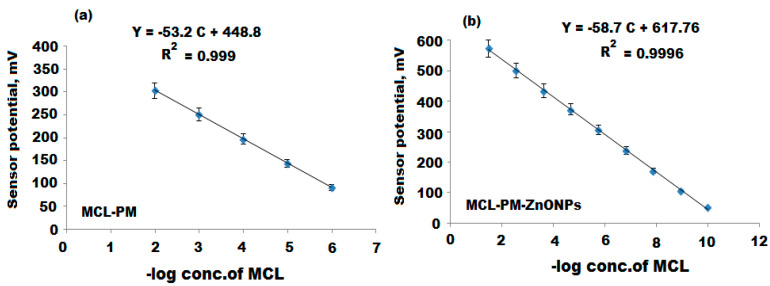
Calibration graphs of the constructed (**a**) Conventional minocycline–phosphomolybdate (MCL-PM) and (**b**) Modified MCL-PM-ZnONPs-coated wire sensors.

Another modified sensor was constructed by covering the surface of the sensor with a thin layer of ZnONPs. The performance characteristics of the constructed sensors were studied with respect to the response time and their sensitivity toward the determination of MCL standard solutions (Table 1). The outcomes revealed that the constructed sensors showed Nernstian responses with slopes of 53.2 ± 0.5 and 58.7 ± 0.2 mV over the drug concentration ranges of 1.0 × 10^−6^–1.0 × 10^−2^ and 1.0 × 10^−10^–1.0 × 10^−2^ mol L^−1^ for conventional MCL-PM and modified MCL-PM-ZnONPs, respectively (Figure 8a,b). The dynamic responses of the two constructed coated wire sensors were recorded to be 60 and 45 s and the lifetimes were 20 and 50 days for the previously mentioned sensors, respectively.

The obtained results indicated that the modified coated sensor using ZnONPs displayed high dynamic response with a wide concentration range detection compared with the non-modified one. As previously reported, nanoparticles have been used to construct various types of chemosensors based on their advanced physical and chemical features, including a large surface area/volume ratio, high conductivity and mechanical strength, and excellent electrocatalytic activity [43]. The exceptional electrical properties of metal oxide nanoparticles and their hydrophobicity, which eliminate the undesired aqueous layer between the electronic conductor and the coated membrane, not only prevent the leaching of active material (ionophore) from the ion-selective membrane to the tested medium but also develop a more durable and an alternative semiconducting sensing membrane [44]. The increase of Nernstian response to 58.7 ± 0.2 mV of MCL-PM-ZnONPs can be attributed to the nanosized zinc oxide inducing a number of free electrons at the surface of the membrane, which in turn enhances both the slope value and sensing behavior of the modified sensor [45].

**Figure 9:**
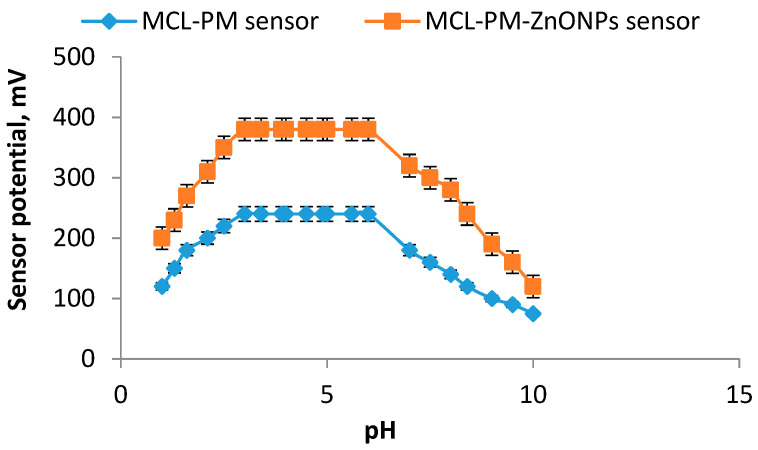
Effect of pH on the constructed conventional and modified ZnONPs-coated wire sensors using 1.0 × 10^−4^ mol L^−1^ of MCL solution.

The performance of the membrane sensors can be greatly influenced by the interference of hydrogen ions. Therefore, it is very important to determine the suitable pH range where the performance of the sensor is not affected by hydrogen ions. The safe pH values of both constructed sensors were tested using 1.0 × 10^−4^ mol L^−1^ of MCL standard solution. Figure 9 shows that the conventional MCL-PM and modified MCL-PM-ZnONPs were practically independent and the potential readings remain constant at pH values in the range from 3 to 5. At lower pH values less than 3, H^+^ ions increased and the potential readings were slightly decreased as a result of the formation of a protonated ion pair that is poorly responsive to MCL ions and has a strong response to hydronium ions in the test solution. However, in alkaline medium at pH value higher than 5, the potential readings were also decreased gradually. The increase of OH^−^ ions cause a competition between MCL ions and OH^−^ ions and consequently decreases the interaction between the ions of the testing drug and the ion-pair sites on the sensor membrane. Thus, the potential responses of the constructed sensors were decreased [46].

In order to study the selectivity of the developed sensors toward the quantification of MCL drug, the constructed sensors were employed to analyze 1.0 × 10^−3^ mol L^−1^ of various interfering species such as inorganic cations, sugars, amino acids, and inactive ingredients, which were added to the oral tables as additives such as talc, ferric dioxide, silicon dioxide, titanium dioxide, magnesium stearate, and microcrystalline cellulose). Both sensors showed high selectivity toward the determination of MCL, whereas the developed MCL-PM-ZnONPs displayed excellent selectivity rather than the conventional MCL-PM sensor. Additionally, the selectivity of MCL-PM and MCL-PN-ZnONPs-coated wire sensors referred to the free energy transfer of MCL^+^ that occurs between the investigated drug solution and the coated membrane phases. Table 2 showed that no interference was observed when using the suggested sensors for the detection of the drug in the presence of the previously mentioned interfering species.

### 3.3. Quantification of Minocycline Hydrochloride

The proposed conventional and modified sensors were used to determine MCL in its bulk form and the calculated percentage recoveries were 99.22 ± 0.6 and 99.57 ± 0.4% for MCL-PM and MCL-PM-ZnONPs, respectively (Table 3). The high sensitivity and good recovery percentage obtained by using the modified sensor were due to the high dielectric constant of ZnONPs, which enhanced the selectivity and elevated the sensitivity of the constructed sensor toward the determination of the tested drug.

### 3.4. Method Validation

The developed electrochemical method for the determination of MCL using conventional MCL-PM and modified MCL-PM-ZnONPs was validated according to International Council for Harmonisation of Technical Requirements for Pharmaceuticals for Human Use ICH guidelines [47]. It was found that both sensors displayed linear relationships over 1.0 × 10^−6^–1.0 × 10^−2^ and 1.0 × 10^−10^–1.0 × 10^−2^ mol L^−1^ for the conventional and modified coated wire sensors, respectively. The regression equations were E_mV_ = (53.2 ± 0.5) log [MCL] + 448.8 and E_mV_ = (58.7 ± 0.2) log [MCL] + 617.76 for conventional and enriched ZnONPs-coated wire sensors, respectively. The correlation coefficients and the lower detection limits were 0.9995 and 0.9998, 4.9 × 10^−7^ and 5.0 × 10^−11^ mol L^−1^ for the previously mentioned sensors, respectively. The accuracy of the proposed method was investigated using various MCL concentrations, and the results were expressed as mean percentage recoveries (99.22 ± 0.6% and 99.57 ± 0.4%) for the developed sensors, respectively. A further study was carried out to ensure the intermediate precision of the suggested method using inter-day and intra-day assay, and the percentage relative standard deviation (% RSD) was determined (Table 4). The calculated % RSD values were 0.3% and 0.8% for intra-day and inter-day assays of MCL-PM-ZnONPs, and the outcomes of the results were less than 2%, confirming a highly precise method. To ensure the robustness of the current method, acetate buffer of pH 5 ± 0.2 was applied, and the percentage recovery was calculated as 99.18 ± 0.3% and 99.48 ± 0.2% for a conventional MCL-PM and modified MCL-PM-ZnONPs, respectively.

A further test was performed to ensure the ruggedness of the current analytical method by using a different model of pH meter (Jenway-3510). The obtained mean percentage recoveries were found to be 99.28 ± 0.6% and 99.54 ± 0.3 for the previously mentioned sensors. The results indicated good agreement with those obtained by the proposed method with no significant changes observed.

### 3.5. Quantification of the Drug in Tablets

The tested MCL was determined in Minocycline hydrochloride^®^ tablets (50 mg/tablet). The overall results were statistically treated using Student’s *t*-test and F-test [48] and then compared with previously reported results obtained from a derivative spectrophotometric method [24]; this method was based on the determination of the absorbance of the drug at 283 nm.

The results of the data (Table 5) indicated an excellent sense of the developed sensors toward the determination of MCL in minocycline hydrochloride tablet solutions and the resulted percentage recoveries were 99.50 ± 0.4 and 99.82 ± 0.2 for the constructed MCL-PM and MCL-PM-ZnONPs, respectively.

## 4. Conclusions

This study described the construction of a new accurate and sensitive modified MCL-PM-ZnONPs-coated wire sensor. The critical performance characteristics of the modified sensor were compared to the conventional type of the same drug. The suggested modifying sensor was designed to be excellent in sensitivity for the detection of the investigated drug over a wide concentration range and low limit of detection. Therefore, the proposed modified sensor can be applied for the routine analysis of minocycline hydrochloride in its pharmaceutical industries.

## Figures and Tables

**Table 1:** Characteristic responses of conventional coated wire MCL-PM and modified MCL-PM-ZnONPs-coated wire sensors.

Parameter	Conventional MCL-PM Coated Wire Sensor	Modified MCL-PM-ZnONPs Coated Wire Sensor
Slope (mV. Decade^−1^)	53.2 ± 0.5	58.7 ± 0.2
Intercept	448.8	617.7
Regression equation	E_mV_ = (53.2 ± 0.5) log [MCL]+448.8	E_mV_ = (58.7 ± 0.2) log [MCL]+617.7
Correlation coefficient, r	0.999	0.9996
Linear range (mol L^−1^)	10 × 10^−6^–1.0 × 10^−2^	1.0 × 10^−10^–1.0 × 10^−2^
LOD (mol L^−1^)	4.9 × 10^−7^	5.0 × 10^−11^
Response time/s	60	30
Working pH range	3–5	3–5
Lifetime/day	20	50
Temperature, °C	25	25
Accuracy (%)	99.22 ± 0.6	99.57 ± 0.4

**Table 2:** Selectivity coefficient (K^Pot^_MCL_^+^) of conventional coated wire MCL-PM and modified MCL-PM-ZnONPs sensors by the separate solution method using 1.0 × 10^−3^ mol L^−1^ MCL.

Interferences	Conventional Coated Wire MCL-PM Sensor (K^pot^_MCL_^+^)	Modified MCL-PM-ZnONPs Sensor (K^pot^_MCL_^+^)
Na^+^	6.2 × 10^−3^	4.7 × 10^−6^
K^+^	2.4 × 10^−3^	3.1 × 10^−4^
Ag^+^	7.7 × 10^−3^	6.1 × 10^−5^
Ni^2+^	1.2 × 10^−3^	9.4 × 10^−6^
Cu^2+^	5.0 × 10^−3^	2.9 × 10^−4^
Zn^2+^	6.9 × 10^−3^	6.2 × 10^−3^
Mg^2+^	3.3 × 10^−3^	9.1 × 10^−4^
Fe^3+^	5.7 × 10^−3^	4.2 × 10^−4^
L-histidine	1.3 × 10^−3^	5.2 × 10^−5^
Ornithine	4.4 × 10^−3^	8.4 × 10^−5^
Glycine	5.6 × 10^−3^	3.3 × 10^−5^
Maize starch	5.1 × 10^−3^	8.7 × 10^−4^
Lactose	1.2 × 10^−3^	2.6 × 10^−5^
Glucose	2.8 × 10^−3^	8.7 × 10^−6^
Talc	3.5 × 10^−3^	6.8 × 10^−4^
SiO_2_	7.1 × 10^−3^	1.8 × 10^−6^
TiO_2_	4.6 × 10^−3^	7.4 × 10^−4^
Magnesium stearate	2.9 × 10^−3^	9.0 × 10^−5^
Microcrystalline cellulose	8.4 × 10^−3^	2.7 × 10^−4^

**Table 3:** Statistical analysis of data obtained from the determination of MCL in bulk powder using conventional MCL-PM and modified MCL-PM-ZnONPs-coated wire sensors.

Conventional MCL-PM Coated Wire Sensor		Modified MCL-PM ZnONPs Coated Wire Sensor	
* Test Solution	% Recovery ± SD	* Test solution	% Recovery ± SD
6.0	100.0 ± 0.1	10.0	99.9 ± 0.4
5.3	99.6 ± 0.3	9.0	99.7 ± 0.2
5.0	99.8 ± 0.3	8.0	100.0 ± 0.6
4.3	98.1 ± 0.7	7.0	99.3 ± 0.7
4.0	98.8 ± 0.5	6.0	99.7 ± 0.9
3.3	98.8 ± 0.2	5.0	100.0 ± 0.4
3.0	99.7 ± 0.9	4.0	99.3 ± 0.1
2.3	98.7 ± 0.5	3.0	98.7 ± 0.8
2.0	99.5 ± 0.3	2.0	99.5 ± 0.3

* Test solution using—log Conc. mol L^−1^.

**Table 4:** Intra-day and inter-day assay of minocycline hydrochloride using a modified MCL-PM-ZnONPs-coated wire sensor.

Modified MCL-PM-ZnONPs Coated Wire Sensor			
Intra-Day Assay		Inter-Day Assay	
* Test Solution	% Recovery ± SD	* Test Solution	% Recovery ± SD
9.0	99.7 ± 0.3	9.0	99.7 ± 0.8
6.0	99.3 ± 0.4	6.0	99.3 ± 1.0
3.0	100.0 ± 0.2	3.0	100.0 ± 0.6

* Test solution using—log Conc. mol L^−1^.

**Table 5:** Statistical analysis of data obtained from the determination of MCL in Minocycline hydrochloride^®^ tablets using conventional MCL-PM and modified MCL-PM-ZnONPs-coated wire sensors.

Conventional MCL-PM Coated Wire Sensor		Modified MCL-PM ZnONPs Coated Wire Sensor		
* Test solution	% Recovery	* Test solution	% Recovery	
6.0	99.7 ± 0.3	7.0	99.4 ± 0.1	Reported Method [24]
5.3	99.2 ± 0.6	6.0	99.7 ± 0.4	
5.0	99.4 ± 0.2	5.0	99.8 ± 0.3	
4.0	100.0 ± 0.1	4.0	100.0 ± 0.1	
3.0	99.7 ± 0.6	3.0	100.0 ± 0.2	
2.0	99.0 ± 0.4	2.0	100.0 ± 0.5	
99.50 ± 0.4 0.700 (2.228) *** 1.78 (5.05) ***		99.82 ± 0.2 1.248 (2.228) *** 2.25 (5.05) ***		99.64 ± 0.36

* Test solution and found using—log Conc. mol L^−1^; *** The tabulated values of “*t*”-test and “*F*”-test at confidence level *p* = 0.05 [48].
